# Factors Associated with Sleep Disruption and Fatigue in Thyroid Cancer Survivors

**DOI:** 10.3390/curroncol33010001

**Published:** 2025-12-19

**Authors:** Domenic DiSanti, Abbey Fingeret, Makayla Schissel, Christopher Wichman, Hannah Coldiron, Oleg Shats, Su Chen, Whitney Goldner

**Affiliations:** 1 Department of Internal Medicine, Divison of Endocrinology, University of Nebraska Medical Center, Omaha, NE 68198, USA; 2 Department of Surgery, University of Nebraska Medical Center, Omaha, NE 68198, USA; 3 Fred and Pamela Buffett Cancer Center, University of Nebraska Medical Center, Omaha, NE 68198, USA; 4 College of Public Health, University of Nebraska Medical Center, Omaha, NE 68198, USA; 5 University of Nebraska Lincoln, Lincoln, NE 68588, USA; 6 Department of Internal Medicine, Division of Endocrinology, Metabolism, Diabetes, University of Colorado, Anschutz Medical Campus, Auora, CO 80045, USA

**Keywords:** thyroid carcinomas, nonmedullary (MeSH ID: D000077273), health-related quality of life (MeSH ID: D011788), fatigue (MeSH ID: D005221), sleep disorder (MeSH ID: D012893), vocal cord paralysis (MeSH ID: D014826), parathyroid disorder (MeSH ID: D010279), female sex (MeSH ID: D005260), Pittsburgh Sleep Quality Index (PSQI), Brief Fatigue Inventory (BFI)

## Abstract

Thyroid cancer survivors often experience worse quality of life than other cancer survivors, with fatigue and sleep disturbance being common contributors. In this prospective cohort from the ICaRe2 cancer registry, survivors completed the Brief Fatigue Inventory (BFI) and Pittsburgh Sleep Quality Index (PSQI) at enrollment and follow-up, with univariate and multivariable analyses identifying factors associated with fatigue and sleep quality. Among 249 survivors (83% female, median age 42), 205 completed the BFI and 224 the PSQI. Most were low (57%) or intermediate (34%) risk or recurrence at diagnosis, and 74% had no structural recurrence. Poor sleep and greater fatigue were significantly associated with female sex (*p* = 0.0003 and 0.001), younger age at diagnosis (*p* = 0.02 and 0.0006), and vocal cord paralysis (*p* = 0.01 and 0.046). Fatigue was also higher in those with hypoparathyroidism (*p* = 0.04). No associations were found with recurrence risk, therapy response, thyroid hormone type, or TSH levels. Younger female survivors, particularly those with vocal cord paralysis or hypoparathyroidism, are more prone to fatigue and poor sleep, highlighting potential targets for interventions to improve quality of life.

## Simple Summary

Thyroid cancer is one of the most common cancers affecting young women, and although survival rates are very high, many survivors struggle with long-term issues that impact their quality of life. Fatigue and poor sleep are especially common, but the pattern of who experiences these problems has not been well understood. In this study of thyroid cancer survivors, we found that women diagnosed at a younger age were more likely to report worse sleep and greater fatigue. Those who developed vocal cord paralysis or hypoparathyroidism were also more likely to experience fatigue and sleep problems. These findings suggest that the challenges thyroid cancer survivors face are not just related to their cancer treatment or hormone levels. Better understanding of the factors that are impacting these young women could help guide future research and develop strategies to improve daily well-being and long-term health in thyroid cancer survivors.

## 1. Introduction

Differentiated thyroid cancer (DTC)—including papillary, follicular, and oncocytic types, accounts for approximately 97% of all thyroid cancers [1]. As of 2020, an estimated 950,000 people in the United States were living with thyroid cancer [2]. Its incidence rose by about 3% annually from 1974 to 2013 before leveling off, a trend also observed in Europe, Australia, and Asia [3]. Notably, over half of U.S. cases are diagnosed in individuals under 55 years old and the disease is about three times more common in females. Given the low mortality rate, the population of thyroid cancer survivors, primarily young women, is steadily growing [4,5].

Prior studies have shown that thyroid cancer survivors report overall lower QoL compared to the general population, as well as those with breast, colorectal, and prostate cancer [6]. It is not fully known which factors contribute to worse QoL in thyroid cancer survivors when compared to more aggressive cancers. However, fatigue and sleep disturbance are common in thyroid cancer survivors and may be drivers of poor QoL. The Pittsburgh Sleep Quality Index (PSQI) and the Brief Fatigue Inventory (BFI) have been used in conjunction with QoL questionnaires to evaluate sleep and fatigue and their impact on QoL in thyroid cancer survivors.

Studies in cancer populations have shown a direct link between impaired sleep quality and QoL [7]. There is significant impairment in sleep quality in the DTC population compared to those with benign thyroid nodules and impaired sleep quality has been reported in as many as 76% of papillary thyroid carcinoma patients [8,9]. DTC patients report higher rates of sleep disturbances than the United States normative population as well as non-Hodgkin lymphoma, breast, colorectal, uterine, and prostate cancer survivors [10,11]. Poorer sleep quality (via PSQI) has been demonstrated in a case-controlled study of those with DTC vs. healthy controls [12]. Poor sleep quality was also significantly correlated with worse QoL in thyroid cancer survivors (QoL assessed via THyPro (measure of thyroid disease-related QoL), suggesting an association between QoL and sleep quality [13].

Increased fatigue has also been reported in up to 42% of DTC survivors [6,10,14,15,16,17]. Patients with thyroid cancer report worse fatigue than those with non-Hodgkin’s lymphoma, breast, colorectal, uterine, and prostate cancer [11]. Self-reported significant fatigue was a predictor of poor overall QoL in DTC survivors when assessing QoL using composite scores from the 36-Item Short Form Survey (SF-36) and THYCA-QoL 24 forms [18,19]. Additionally, Lee et al. reported that increased fatigue was significantly correlated with lower QoL scores in disease-free thyroid cancer survivors utilizing both the BFI and EORTC QLQ-C30 QOL questionnaire [20].

In this study, we sought to specifically evaluate factors that impact fatigue and sleep disturbance in thyroid cancer survivors to better understand the contributors to impaired QoL of this population.

## 2. Materials and Methods

### 2.1. Recruitment

This is a prospective longitudinal cohort analysis of patients with DTC. This is a longitudinal cohort design so that quality of life could be assessed at enrollment and over time. People with DTC who voluntarily enrolled into the integrated Cancer Repository for Cancer Research (ICaRe2) registry at the Fred and Pamela Buffett Cancer were included in the study. ICaRe2 is an IRB approved (approved number: 253-13; approved date: 24 May 2019) bioinformatics and biospecimen registry for adults with cancer or who are at high risk for developing cancer. The Thyroid Cancer Registry is part of the ICaRe2 registry. All patients with thyroid cancer are invited to enroll in this registry at any point after diagnosis. Patients who developed recurrence during enrollment were included in the dataset, as well as those enrolled for the first time due to newly identified recurrence. The registry was started in July 2008 and data collection is ongoing. For this study, data collection ended in January 2022. This registry is open to anyone with thyroid cancer, but only patients with DTC were included in the analysis for this study. Patients aged 18 years or older at the time of diagnosis were eligible. Surgical treatment modalities included lobectomy, thyroidectomy, thyroidectomy with neck dissection (surgical procedure in which lymph nodes from specific neck compartments are systematically removed to treat confirmed or suspected locoregional nodal metastases from thyroid cancer), or neck dissection following thyroidectomy. Patients may have received no radioactive iodine (RAI), a single dose, or multiple treatments with varying doses. For those who underwent RAI, preparation could have involved either thyroid hormone withdrawal or Thyrogen stimulation. Patients with medullary thyroid carcinoma, struma ovarii, or anaplastic histology were excluded given their underlying disease was not DTC. Since the questionnaires were only in English at the inception of the registry, only English-speaking patients were included. Most patients were enrolled into the registry after initial surgery when final pathology was available. All patients were provided with informed consent to participate in the registry as well as future studies using registry data. Informed consent and enrollment were conducted in person; questionnaires were sent to patients’ personal email addresses and completed using internet-enabled devices in a non-clinic setting. Participants enrolled in the database between 2008 and 2020 completed the PSQI at the time of enrollment. Beginning in 2020, all previously enrolled participants were invited to complete the BFI for the first time. Participants enrolling in 2020 or later were asked to complete both the PSQI and BFI at the time of enrollment. All participants who had already completed the enrollment surveys between 2008 and 2020 were asked to complete a follow-up survey if it had been at least six months since the last survey completion. Most patients included in the study filled out the initial questionnaire, and a smaller proportion filled out two questionnaires as noted below.

Participants were also asked to complete a demographic, family history, and personal medical questionnaire as part of the registry. Clinical variables specific to thyroid cancer were retrospectively obtained from the medical record corresponding to the time of each survey to account for changes in clinical status and treatment from enrollment to follow-up. Clinical variables include histology, TNM, AJCC 8th Edition stage, type of surgery, RAI, type of thyroid hormone therapy, labs, and side effects of treatment. The American Thyroid Association 2015 Risk of Recurrence was assessed at diagnosis, and Response to Therapy was performed at each follow up visit. Labs including TSH, thyroglobulin, and thyroglobulin antibodies were also obtained at the time of study questionnaire.

### 2.2. Pittsburgh Sleep Quality Index (PSQI)

The PSQI is a self-administered survey used to assess quality of sleep over the previous week. It is a 19-question tool, with seven composite scores regarding sleep quality including global subjective sleep quality (PSQI-Global), sleep duration (PSQI-DURAT), sleep disturbance (PSQI-DISTB), sleep latency (PSQI-LATEN), subjective sleep quality (PQSI-SQUAL) daytime dysfunction (PSQI-DAYDYS), sleep efficiency (PSQI-HSE), and sleep medication usage (PSQI-MEDS). PSQI-Global can range from 0–21 and is obtained by summing the above composite scores. A global score of ≤5 suggests a “good sleeper” and those with scores > 5 “bad sleepers”. Consent to use this questionnaire was obtained to be included in the ICaRe2 registry. We also used the magnitude of the PSQI global score to compare across populations in this study.

### 2.3. Brief Fatigue Inventory (BFI)

The BFI is a self-administered fatigue survey used to assess patients’ function over the last 24 h. The survey consists of nine items that can be completed within 2–3 min. Scoring is made on each individual item from a scale of 0–10 where 0 means “no fatigue” and 10 meaning “fatigue as bad as you can imagine”. Global fatigue scores can be calculated by averaging the scores obtained in each of the nine items [21]. Consent to use this questionnaire was obtained to be part of the ICaRe2 registry.

### 2.4. AJCC 8th Edition/Risk of Recurrence and Response to Therapy

The ICaRe2 registry was initiated in 2008 while the AJCC 7th edition TNM staging was standard of care, but the current standard is the AJCC 8th edition [22]. The enrollment and data collection spans the time frame using both the 7th and 8th edition, so to be consistent in our analysis using AJCC staging, we staged each person according to the 8th edition for the analysis regardless of their year of diagnosis [23,24]. Each participant was also assessed for American Thyroid Association Risk of Recurrence at time of diagnosis and Response to Therapy at time of questionnaire completion. Patients were enrolled into the registry beginning in 2008, spanning the timeframe both the 7th and 8th edition AJCC staging was used as standard of care. Furthermore, at the time of data collection and analysis, the American Thyroid Associations Risk of Recurrence and Response to Therapy guidelines were based on the 2015 guidelines. As of recently, a 2025 update to these guidelines has been published [1,25]. Hence, Response to Therapy and Risk of Recurrence in this study are specifically referencing the 2015 guidelines.

### 2.5. Overall Statistical Analysis

This analysis included a total of 249 DTC patients. Descriptive statistics (mean, standard deviation, median, IQR, range, counts, and percentages) were used to summarize patient characteristics at their first survey. To assess associations between demographic, clinical, and treatment variables and the PSQI and BFI global score, mixed-effects models were used. The PSQI or BFI global scores served as the outcome in the model, and demographic, clinical, and treatment variables were used as predictor variables. Repeated measures were specified by the survey number and nested within each participant’s time-dependent predictors. These included age at diagnosis, time since diagnosis, total number of procedures prior to survey, time since most recent procedure or radioactive iodine, and number of procedures or radioactive iodine prior to survey. Age at time of survey were modeled the same as above, with the addition of random intercept for each subject instead of specifying repeated measures for survey number. Both repeated measures and random intercept approaches previously described included survey numbers as a fixed effect in the models. The survey number was calculated individually for the BFI and PSQI questionnaires, as the global scores were analyzed independently. To account for patients who had both the enrollment survey and the follow-up survey, an autoregressive covariance matrix was selected. For predictors that had issues with convergence, an unstructured covariance structure was used. Model estimated means were generated to show the adjusted mean outcome for each group for categorical variables, or if the variable was continuous, adjusted mean outcomes were calculated for three example values across the range for that continuous variable.

### 2.6. Additional PSQI/BFI Statistical Methods

#### 2.6.1. Stratification by Age at Survey

Since thyroid cancer is more common in women and occurs at a younger age, we wanted to evaluate for the possible confounder of sleep disruption and fatigue associated with menopause. To adjust for this, PSQI and BFI models exploring gender were further investigated by stratifying by age at survey into three groups women across three age ranges; those < 45 years, those 45–55 years, and those > 55. These models were also adjusted for survey numbers and used an autoregressive covariance structure as some individuals had more than one survey within these age ranges.

#### 2.6.2. Multivariable Analysis with Age at Survey and Gender

Multivariable mixed-effect models including age at survey group (<45 years, 45–54 years, and 55+ years), gender, and their interaction as predictors were performed for both the PSQI and BFI global outcome scores. These models also included survey numbers as a fixed effect and utilized the random intercept structure previously described. These BFI and PSQI multivariable models were executed using a random intercept for each subject.

## 3. Results

### 3.1. Demographics at Time of First Survey

A total of 249 participants completed surveys: 205 (82.3%) completed the Brief Fatigue Inventory (BFI) and 224 (90%) completed the Pittsburgh Sleep Quality Index (PSQI) at enrollment. Beginning in 2020, all previously enrolled participants were invited to complete the BFI for the first time. Participants enrolling in 2020 or later were asked to complete both the PSQI and BFI at the time of enrollment

For longitudinal follow-up:

- 73 participants (29.3%) completed a second BFI survey;
- 132 participants (53%) completed a second PSQI survey.

For the 132 participants completing two PSQI surveys, the median (IQR) time between surveys was 115.5 months (10.5 to 144.5). For the 73 participants completing two BFI surveys, the median (IQR) interval was 8.0 months (8.0 to 9.0).

Overall, 83% were female and 95% were non-Hispanic White. The median age at diagnosis was 42 years (IQR 12 to 85). Baseline demographics, TNM and AJCC 8th Edition stage, ATA risk of recurrence category, treatment, ATA response to therapy, and long-term side effects are summarized in Table 1.

### 3.2. PSQI Global Score Analysis

#### 3.2.1. Univariate PSQI Global Score

In the PSQI survey, higher scores indicate poorer sleep quality.

Supplementary data of patients characteristics and PSQI is seen in Supplmentary Table S2.

- Younger age at diagnosis was associated with higher PSQI scores (*p* = 0.02).
- Females had significantly higher PSQI scores than males (*p* = 0.0003).
- Participants with vocal cord paralysis had worse scores than those without (*p* = 0.01).

No significant associations were found with extent of surgery, number of procedures, RAI therapy, TNM stage, ATA risk of recurrence, TSH level, or type of thyroid hormone therapy. Vocal cord paralysis (*p* = 0.01) remained the most significant clinical factor affecting sleep quality (Table 2).

#### 3.2.2. Stratified PSQI Global Score by Age at Survey

After adjusting for survey number, females <45 years had significantly higher model-adjusted mean PSQI scores (9.69 ± 0.35) compared to males (7.13 ± 0.99, *p* = 0.02), suggesting poorer sleep quality among younger females (Table 3).

#### 3.2.3. Multivariable PSQI Global Score: Age at Survey and Gender

After adjusting for survey number, age group, and age-by-gender interaction, females continued to have worse sleep quality (9.00 ± 0.23) than males (7.07 ± 0.54, *p* = 0.001). This difference persisted in multivariable analysis (Table 4).

### 3.3. BFI Global Score Analysis

#### 3.3.1. Univariate BFI Global Score

In the BFI survey, higher scores indicate greater fatigue.

Supplementary data of patients characteristics and BFI is seen in Supplmentary Table S1.

- Fatigue decreased with increasing age at diagnosis: 30 years (3.21 ± 0.22), 40 years (2.90 ± 0.17), and 50 years (2.59 ± 0.19, *p* = 0.01).
- Fatigue also decreased with increasing age at survey: 45 years (3.08 ± 0.19), 50 years (2.86 ± 0.16), and 55 years (2.65 ± 0.16, *p* < 0.01).
- Females had significantly higher mean global BFI scores (2.98 ± 0.18) than males (1.64 ± 0.37, *p* = 0.001).
- Participants with vocal cord paralysis had higher fatigue (4.95 ± 1.11) than those without (2.69 ± 0.17, *p* = 0.04).
- Fatigue was also higher among those with hypoparathyroidism (*p* = 0.04).
- No significant associations were found with number of procedures, RAI therapy, ATA risk of recurrence, response to therapy, TSH level, or type of thyroid hormone therapy (Table 5).

#### 3.3.2. Stratified BFI Global Score by Age at Survey

Model-adjusted mean global BFI scores were significantly higher in females (3.4 ± 0.36) compared to males (0.72 ± 0.99, *p* = 0.01) in the 45–54 years age group, suggesting greater fatigue in midlife female survivors (Table 6).

#### 3.3.3. Multivariable BFI Global Score: Age at Survey and Gender

In multivariable analysis adjusting for survey number and age group, females continued to report greater fatigue (3.08 ± 0.18) than males (1.48 ± 0.44, *p* = 0.001), confirming gender as an independent predictor of fatigue (Table 7).

## 4. Discussion

In this study, we found that females who were diagnosed with thyroid cancer at a younger age had worse sleep and higher rates of fatigue. This suggests that aggressiveness of disease and residual or recurrent disease does not play a significant role in sleep or fatigue. We did not see any association with other clinical factors such as initial ATA Risk of Recurrence, or Response to Therapy. The Risk of Recurrence and Response to Therapy were based on the ATA 2015 Thyroid Cancer Guidelines [1]. The specific criterion from the guidelines is placed in the supplement for review (Supplemental Tables S3 and S4). Additionally, we did not find an association between thyroid hormone therapy or TSH levels, which was unexpected. We hypothesized that lower TSH could contribute to poor sleep since subclinical or overt hyperthyroidism has the potential to cause sleep disturbance and decreased energy levels, but we did not see a significant association. Iatrogenic hypoparathyroidism was found to be associated with increased rates of reported fatigue, likely due to fatigue secondary to chronic hypocalcemia and neuromuscular instability (cramping, and oral numbness). Those on levothyroxine monotherapy also did not have significantly worse scores than those on T3/T4 combination therapy, indicating type of therapy was not significantly associated. There were no significant associations with other side effects including sialadenitis, xerostomia, or lacrimal duct obstruction or type and number of surgeries in this study, but this observation warrants further investigation especially the conflicting data on QoL and with types of surgery [26,27,28]. Notably, this study was conducted prior to the ATA 2025 DTC Guidelines, as such active surveillance was not implemented as a management modality in this population and would be of particular interest in future studies regarding QoL in this population [25].

Worse sleep quality in thyroid cancer patients compared to the general population and benign thyroid nodules has been demonstrated [8,9,13]. In this study, we found that females, overall, had significantly worse sleep quality than males and those who were younger age at diagnosis also reported worse sleep quality. This is not the first-time female sex has been associated with worse sleep quality in a thyroid cancer population. Rodger et al. also found a higher reported rate of insomnia in females compared to males in the DTC population [29]. Zhang et al. showed that female sex was correlated with worse sleep quality using the PSQI in DTC patients compared to healthy controls in their univariate analysis; however this was not seen in their multivariate analysis [12]. In our study, females had worse sleep quality in both univariate and multivariable analysis.

The presence of vocal cord paralysis was also significantly associated with sleep disturbance and fatigue. It is known that people with vocal cord paralysis often have increased shortness of breath with activity and exertion and require increased effort to talk and raise vocal volume. Additionally, post-operative hypoparathyroidism was also associated with fatigue. This is consistent with a study from Chen et al. who also reported worse QoL in those with surgical hypoparathyroidism [30]. In contrast to our study, Chen also reported significant associations between QoL and higher cumulative RAI, history of lateral neck dissection, lower income, and conventional surgery [30].

Like our findings with impaired sleep, we also found a significant association between younger age at diagnosis, female sex, and patient-reported fatigue. DTC survivors and relationships to increased fatigue, female sex, and age have been demonstrated in previous studies supporting our findings; however, this population was composed of exclusively those without persistent or recurrent disease [18]. Younger age at the time of thyroid cancer diagnosis and increased fatigue has been demonstrated in both the young adult and pediatric populations (pediatric patients were excluded in the current study) [31,32]. Our study did not evaluate degree of physical activity achieved by patients. Studies have shown physical activity can improve fatigue in DTC patients following thyroidectomy [33,34]. In light of this, future studies should attempt to quantify physical activity level and its relationship to patient-reported fatigue in this population.

There are many factors that can impact symptoms of fatigue. Notably, retrospective analyses have demonstrated that female sex is a risk factor for cancer-related fatigue, which we are further supporting by our findings [35,36]. Certainly, poor sleep quality can result in increased fatigue; hence, the PSQI and BFI may be capturing similar data or capturing fatigue because of poor sleep quality. However, there could be other causes of fatigue. Depression and anxiety are known to impact fatigue and sleep quality. Choi et al. reported 8.9% of patients who undergo thyroid surgery go on to develop major depressive disorder [37]. Because thyroid cancer is associated with low mortality rates, thyroid cancer survivors often report being told they have a “good cancer”. This can lead to feelings of not being taken seriously, increased frustration if they experience a recurrence of cancer, and even feeling a lack of validation over their cancer journey [38]. Additionally, the emotional impact of fear of recurrence, which has been demonstrated to impair QoL in studies in thyroid cancer patients, was not directly evaluated in our study [39]. All these potential associations need to be further explored but could be contributors to increased fatigue in this population.

The impact of this work is primarily exploratory; however, being able to identify young female patients who may be having increased fatigue and sleep quality as part of their thyroid cancer treatment can be of utility to the practicing clinician. It will allow physicians to empathize with their patients and the symptoms they may be experiencing and together can engage in discussions to validate their cancer experience. Impacts of sleep hygiene, physical activity, and cognitive behavioral therapy regarding fatigue, sleep quality, and QoL in the DTC could be explored in future research.

### Limitations

This study population was predominantly white and approximately 25% of participants had incomplete RAI data due to treatment from outside institutions. Regarding sleep quality, our patient demographics did not account for night swift workers or those with irregular sleep schedules which may impact results. Some data in the analysis was also collected during the COVID-19 pandemic, possibly influencing responses. All these place limitations on the current analysis.

Incomplete date variables were common, and in such cases January was used for missing months, and the 1st of each month was used if missing the date. While this remains a limitation, given the follow-up periods in the analysis often spanned years, we do not feel this decision has significantly impacted the results. Not all patients completed every QOL questionnaire, and response rates declined over time. The BFI, introduced in late 2020, had a shorter follow-up period (8 months vs. 115.5 months for PSQI) and lower response rate, likely due to its staggered implementation. This staggered collection does limit the longitudinal interpretation of fatigue data, as several patients were further out from their initiation surgery at time first completing this item. However, in the analysis, there was still no significant difference from time from diagnosis for those who were collected closer to their diagnosis date. Incomplete covariate data further limited the number of usable responses in the models. Furthermore, psychological and mood factors such as anxiety, depression, and acute stress were not accounted in the data collection limiting the causal interpretation of these results.

## 5. Conclusions

Female sex and younger age thyroid cancer survivors report worse sleep and greater fatigue, which are likely key contributors to reduced quality of life. While complications such as vocal cord paralysis and hypoparathyroidism predict fatigue and poor sleep, most clinical variables do not. Broader use of thyroid-specific and general QoL tools may help identify the multifactorial drivers of reduced QoL and guide targeted interventions.

## Figures and Tables

**Table 1:** Patient demographics.

	n = 249
Female, n (%)	206 (82.7)
Race, n (%)	
Non-Hispanic White	236 (95)
Other	134 (5)
Age at Diagnosis (median (IQR))	41.9 (3 to 52.7)
Age at Survey (median (IQR))	47 (38 to 57)
Time Since Diagnosis (median (IQR))	49 (8 to 93)
Histology, n (%)	
Follicular/Papillary	232 (96.6)
Oncocytic	17 (4)
Number of Procedures (median (IQR))	1 (1 to 2)
Time Since Last Procedure (median months (IQR))	53 (21 to 91)
Number of RAI (median (IQR))	1 (1 to 1)
Time Since Last RAI (median months (IQR))	58 (25 to 140)
AJCC 8th Edition Staging T Stage, n (%)	
T1	130 (53.7)
T2	73 (30.2)
T3/T4	39 (16.1)
AJCC 8th Edition N Stage, n (%)	
N0	154 (62.1)
N1a	44 (17.7)
N1b	50 (20.2)
AJCC 8th Edition M Stage	
M0	237 (95.6)
M1	11 (4.4)
AJCC Overall Staging 8th Edition n (%)	
I	221 (89.5)
II	18(7.3)
III	4 (1.6)
IV	4 (1.6)
ATA Risk of Recurrence, n (%)	
Low	140 (56.7)
Intermediate	85 (34.4)
High	22 (8.9)
Actual Recurrence Requiring Additional Surgery or RAI, n (%)	64 (25.8)
ATA Response to Therapy, n (%)	
Excellent	148 (59.7)
Indeterminate	72 (29)
Biochemically Incomplete	18 (7.3)
Structurally Incomplete	10 (4)
TSH Categorical, n (%)	
<0.1	44 (17.7)
0.1–0.29	71 (28.6)
0.5–1.9	70 (258.2)
>2	63 (25.4)
TSH (median (IQR))	0.6 (0.2 to 2.0)
Thyroid Hormone Replacement	
Levothyroxine Monotherapy	224 (90.7)
T4 + T3 (separate prescriptions)	21 (8.5)
T4 + T3 (combined, desiccated)	2 (0.8)
Thyroglobulin (median (IQR))	0.1 (0.1 to 0.6)
Tg Ab +	13 (5.2%)
Side Effects of Therapy, n (%)	
Sialoadenitis	41 (16.5)
Lacrimal Duct Obstruction	13 (5.2)
Xerostomia	46 (18.5)
Neck Pain	8 (3.2)
Hoarseness or Voice Changes	18 (7.3)
Vocal Cord Paralysis	4 (1.6)
Hypoparathyroidism	24 (9.4)

Legend: RAI: radioactive iodine, AJCC Joint Committee of Cancer, ATA: American Thyroid Association (Risk or Recurrence and Response to Therapy Criteria can be found in Supplemental Tables S3 and S4), TSH: thyroid stimulating hormone, Tg Ab +: thyroglobulin antibody positive.

**Table 2:** Univariate PSQI global score.

		N of Surveys Analyzed	Model-Adjusted Mean	Standard Error	Type III *p*-Value
Age at Diagnosis (continuous) *		336			**0.02**
	30 years		8.68	0.30	
	40 years		8.32	0.24	
	50 years		7.97	0.27	
Time Since First Diagnosis (continuous) *		356			0.12
	36 months		8.37	0.25	
	96 months		8.17	0.23	
	156 months		7.96	0.29	
Number of Procedures Before Survey (continuous) *		356			0.12
	0		8.73	0.39	
	2		7.94	0.29	
	4		7.16	0.72	
Time Since Last Procedure (continuous) *		332			0.28
	36 months		8.29	0.26	
	96 months		8.13	0.24	
	156 months		7.98	0.30	
Number of RAIs Before Survey (continuous) *		252			0.58
	0		8.38	0.42	
	2		8.07	0.36	
	4		7.76	0.84	
Time Since Last RAI (continuous) *		227			0.19
	36 months		7.95	0.30	
	96 months		8.06	0.29	
	156 months		8.18	0.30	
**T-Stage**		349			0.62
	T1		8.87	0.31	
	T2		8.44	0.39	
	T3/T4		8.44	0.52	
**N-Stage**		355			0.80
	N0/Nx		8.67	0.28	
	N1a		8.79	0.52	
	N1b		8.36	0.47	
**M-Stage**		355	8.68	0.23	0.22
	M0		7.38	1.03	
	M1				
**AJCC 8th Stage**		353			0.47
	1		8.74	0.24	
	2		7.75	0.78	
	3		8.69	1.59	
	4b		6.87	1.74	
**Gender**		356			**0.0003**
	Female		8.96	0.23	
	Male		6.94	0.50	
Age at Survey (continuous) *		356			**0.003**
	45 years		8.36	0.23	
	50 years		8.13	0.23	
	55 years		7.90	0.25	
**ATA Recurrence Risk**		355			0.18
	High		7.67	0.71	
	Intermediate		9.06	0.37	
	Low		8.49	0.29	
**Experienced Recurrences or Required Additional Treatment**		355			0.68
	No		8.67	0.26	
	Yes		8.47	0.42	
**ATA Response to Therapy**		355			0.44
	Indeterminate		9.04	0.36	
	Biochemical incomplete		8.81	0.68	
	Excellent		8.46	0.26	
	Structurally incomplete		7.88	1.02	
**TSH (categorical)**		355			0.92
	<0.1		8.78	0.43	
	0.1–0.49		8.67	0.34	
	0.5–2.0		8.63	0.33	
	>2.0		8.44	0.36	
TSH (continuous) *		355			0.30
	0.1		8.18	0.24	
	0.5		8.19	0.24	
	2.0		8.20	0.24	
**Thyroid Hormone Therapy**		355			0.31
	Levothyroxine monotherapy		8.49	0.23	
	Levothyroxine + liothyronine		9.59	0.66	
	Combined levothyroxine/liothyronine		13.37	3.46	
**TG (categorical)**		310			0.76
	0–0.19		8.64	0.28	
	0.2–0.99		8.92	0.41	
	1.0–4.99		9.00	0.60	
	5.0–9.99		9.71	1.36	
	10.0+		7.75	1.11	
TG (continuous) *		310			0.20
	0.2		8.44	0.25	
	1.0		8.44	0.25	
	5.0		8.43	0.25	
**TG Ab+ **		355			0.74
	No		8.63	0.22	
	Yes		8.19	1.02	
**Sialadenitis**		355			0.38
	No		8.70	0.24	
	Yes		8.20	0.52	
**Tear Duct Blockage or Tearing**		355			0.09
	No		8.70	0.23	
	Yes		6.98	0.98	
**Dry Mouth**		355			0.89
	No		8.63	0.24	
	Yes		8.55	0.52	
**Neck Pain**		355			0.97
	No		8.61	0.22	
	Yes		8.66	1.25	
**Hoarseness or Voice Changes**		355			0.46
	No		8.57	0.23	
	Yes		9.20	0.83	
**Vocal Cord Paralysis**		355			**0.01**
	No		8.53	0.22	
	Yes		12.50	1.53	
**Hypoparathyroidism**		355			0.22
	No		8.52	0.23	
	Yes		9.40	0.68	

* Continuous variables were modeled linearly and adjusted for survey number and these associations are represented using the mean of the outcome; for example, values of the continuous variable, while holding survey number constant at the first survey. Note: Each model only contains one variable which has been adjusted for survey number. Legend: RAI: radioactive iodine, AJCC: Joint Committee of Cancer, ATA: American Thyroid Association (Risk or Recurrence and Response to Therapy Criteria can be found in Supplemental Tables S3 and S4), TSH: thyroid stimulating hormone, Tg Ab+: thyroglobulin antibody positive.

**Table 3:** Stratified PSQI global score by age at survey.

		N of Surveys Analyzed	Model-Adjusted Mean	Standard Error	Type III *p*-Value
**<45 years**	**Gender**	139			**0.02**
	Female		9.69	0.35	
	Male		7.13	0.99	
**45–54 years**	**Gender**	80			0.19
	Female		9.50	0.44	
	Male		7.96	1.09	
**55+ years**	**Gender**	137			0.08
	Female		7.91	0.37	
	Male		6.63	0.63	

**Table 4:** Multivariable PSQI global score, age at survey and gender.

		N of Surveys Analyzed	Model-Adjusted Mean	Standard Error	Type III *p*-Value
**Age at Survey (categorical)**		356			0.20
	<45 years		8.43	0.51	
	45–54 years		8.22	0.52	
	55+ years		7.46	0.35	
**Gender**					**0.001**
	Female		9.00	0.23	
	Male		7.07	0.54	
**Age at Survey Gender Interaction**					0.66
	<45, Female		9.56	0.34	
	<45, Male		7.31	0.95	
	45–54, Female		9.32	0.39	
	45–54, Male		7.13	0.97	
	55+, Female		8.13	0.35	
	55+, Male		6.78	0.61	

**Table 5:** Univariate BFI global score.

		N of Surveys Analyzed	Model-Adjusted Mean	Standard Error	Type III *p*-Value
Age at Diagnosis (continuous) *		263			**0.01**
	30 years		3.21	0.22	
	40 years		2.90	0.17	
	50 years		2.59	0.19	
Time Since First Diagnosis (continuous) *		278			0.13
	36 months		2.99	0.24	
	96 months		2.84	0.18	
	156 months		2.69	0.16	
Number of Procedures Before Survey (continuous) *		278			0.96
	0		2.71	0.36	
	2		2.73	0.21	
	4		2.75	0.60	
Time Since Last Procedure (continuous) *		277			0.20
	36 months		2.94	0.24	
	96 months		2.80	0.18	
	156 months		2.66	0.17	
Number of RAIs Before Survey (continuous) *		184			0.54
	0		2.58	0.40	
	2		2.89	0.27	
	4		3.20	0.71	
Time Since Last RAI (continuous) *		181			0.42
	36 months		2.93	0.25	
	96 months		2.89	0.22	
	156 months		2.83	0.21	
**T-Stage**		274			0.1
	T1		2.65	0.23	
	T2		2.52	0.30	
	T3/T4		3.54	0.41	
**N-Stage**		277			0.88
	N0/Nx		2.75	0.21	
	N1a		2.81	0.39	
	N1b		2.57	0.36	
**M-Stage**		277			0.23
	M0		2.77	0.17	
	M1		1.87	0.73	
**AJCC 8th Stage**		275			0.94
	1		2.75	0.18	
	2		2.76	0.57	
	3		2.39	1.34	
	4b		1.99	1.36	
**Gender**		278			**0.001**
	Female		2.98	0.18	
	Male		1.64	0.37	
Age at Survey (continuous) *		278			**0.0006**
	45 years		3.08	0.19	
	50 years		2.86	0.16	
	55 years		2.65	0.16	
**ATA Recurrence Risk**		276			0.24
	High		2.82	0.54	
	Intermediate		3.07	0.28	
	Low		2.49	0.22	
**Experienced Recurrences or Required Additional Treatment**		277			0.75
	No		2.71	0.19	
	Yes		2.83	0.32	
**ATA Response to Therapy**		277			0.58
	Indeterminate		2.79	0.26	
	Biochemical incomplete		2.75	0.67	
	Excellent		2.79	0.19	
	Structurally incomplete		1.52	0.87	
**TSH (categorical)**		277			0.47
	<0.1		3.08	0.38	
	0.1–0.49		2.61	0.24	
	0.5–2.0		2.92	0.24	
	>2.0		2.58	0.28	
TSH (continuous) *		277			0.23
	0.1		2.70	0.17	
	0.5		2.70	0.17	
	2.0		2.72	0.16	
**Thyroid Hormone Therapy**		277			0.57
	Levothyroxine monotherapy		2.70	0.18	
	Levothyroxine + liothyronine		*3.09*	0.47	
	Combined levothyroxine/liothyronine		*--*	--	
**TG (categorical)**		238			0.57
	0–0.19		2.86	0.21	
	0.2–0.99		2.86	0.32	
	1.0–4.99		2.72	0.47	
	5.0–9.99		4.91	1.52	
	10.0+		1.96	0.94	
TG (continuous) *		238			0.45
	0.2		2.83	0.18	
	1.0		2.83	0.18	
	5.0		2.83	0.18	
**Tg Ab +**		277			0.72
	No		2.75	0.17	
	Yes		2.45	0.64	
**Sialadenitis**		277			0.35
	No		2.68	0.18	
	Yes		3.08	0.40	
**Tear Duct Blockage or Tearing**		277			0.57
	No		2.72	0.17	
	Yes		3.15	0.74	
**Dry Mouth**		277			0.49
	No		2.69	0.18	
	Yes		2.99	0.39	
**Neck Pain**		277			0.71
	No		2.73	0.17	
	Yes		3.11	1.01	
**Hoarseness or Voice Changes**		277			0.06
	No		2.65	0.17	
	Yes		3.81	0.59	
**Vocal Cord Paralysis**		277			**0.046**
	No		2.69	0.17	
	Yes		4.95	1.11	
**Hypoparathyroidism**		277			**0.04**
	No		2.63	0.17	
	Yes		3.79	0.52	

* Continuous variables were modeled linearly and adjusted for survey number and these associations are represented using the mean of the outcome; for example, values of the continuous variable, while holding survey number constant at the first survey. Note: Each model only contains one variable which has been adjusted for survey number. Legend: RAI: radioactive iodine, AJCC: Joint Committee of Cancer, ATA: American Thyroid Association (Risk or Recurrence and Response to Therapy Criteria can be found in Supplemental Tables S3 and S4), TSH: thyroid stimulating hormone, Tg Ab+: thyroglobulin antibody positive.

**Table 6:** Stratified BFI global score by age at survey.

		N of Surveys Analyzed	Model-Adjusted Mean	Standard Error	Type III *p*-Value
**<45 years**	**Gender**	90			0.10
	Female		3.62	0.32	
	Male		2.18	0.82	
**45–54 years**	**Gender**	56			**0.01**
	Female		3.40	0.36	
	Male		0.72	0.99	
**55+ years**	**Gender**	132			0.18
	Female		2.34	0.25	
	Male		1.68	0.42	

**Table 7:** Multivariable BFI global score. Age at survey and gender.

		N of Surveys Analyzed	Model-Adjusted Mean	Standard Error	Type III *p*-Value
**Age at Survey (categorical)**		278			0.09
	<45 years		2.99	0.41	
	45–54 years		1.87	0.52	
	55+ years		1.98	0.26	
**Gender **					**0.001**
	Female		3.08	0.18	
	Male		1.48	0.44	
**Age at Survey** * * **Gender Interaction **					0.21
	<45, Female		3.73	0.29	
	<45, Male		2.25	0.77	
	45–54, Female		3.21	0.34	
	45–54, Male		0.54	0.99	
	55+, Female		2.31	0.26	
	55+, Male		1.66	0.44	

## Data Availability

The original contributions presented in this study are included in the article/supplementary material. Further inquiries can be directed to the corresponding authors.

## Supplementary Materials

The following supporting information can be downloaded at https://www.mdpi.com/article/10.3390/curroncol33010001/s1, Table S1: BFI: Comparison of clinical characteristics at first survey between patients who completed the baseline survey only versus the baseline and follow-up survey. Table S2: PSQI: Comparison of clinical characteristics at first survey between patients who completed the baseline survey only versus the baseline and follow-up survey. Table S3: ATA 2015 Risk of Recurrence Criteria. Table S4: ATA 2015 Response to Therapy Criteria.

## Abbreviations

The following abbreviations are used in this manuscript:

BFI	Brief Fatigue Inventory
PSQI	Pittsburgh Sleep Quality Index
DTC	Differentiated thyroid cancer
ATA	American Thyroid Association
RAI	Radioactive Iodine
TMN	Tumor, Node, Metastasis
AJCC	American Joint Committee on Cancer
IQR	Interquartile Range
TSH	Thyroid-Stimulating Hormone
