# Supplementary material for: Factors Associated with Sleep Disruption and Fatigue in Thyroid Cancer Survivors
Source: Curr Oncol. 2025 Dec 19;33(1):1. doi: 10.3390/curroncol33010001 (PMC12840390; doi:10.3390/curroncol33010001)
Supplement: Supplementary file 1 [file curroncol-33-00001-s001.zip › curroncol-3961995-supplementary.pdf]

Supplementary Materials:

Supplementary Table S1: BFI: Comparison of clinical characteristics at first survey between patients who completed the baseline survey only versus the baseline and follow-up survey

	Number of Surveys			
	1 (N=132)	2 (N=73)	Total (N=205)	P-value
**Age at Diagnosis**				0.05[1]
N (Missing)	117 (15)	73 (0)	190 (15)	
Mean (SD)	43.8 (14.52)	39.7 (13.07)	42.2 (14.09)	
Median (IQR)	42.8 (33.2, 56.0)	37.9 (30.9, 46.9)	41.6 (31.4, 52.7)	
Range	12.2, 85.0	15.7, 78.3	12.2, 85.0	
**Time since First Diagnosis**				0.84[2]
N (Missing)	132 (0)	73 (0)	205 (0)	
Mean (SD)	140.7 (90.80)	142.9 (99.68)	141.5 (93.82)	
Median (IQR)	133.0 (71.5, 191.5)	132.0 (65.0, 193.0)	132.0 (67.0, 193.0)	
Range	1.0, 581.0	13.0, 520.0	1.0, 581.0	
**Number of Procedures before Survey**				0.64[2]
N (Missing)	132 (0)	73 (0)	205 (0)	
Mean (SD)	1.5 (0.77)	1.4 (0.62)	1.4 (0.72)	
Median (IQR)	1.0 (1.0, 2.0)	1.0 (1.0, 2.0)	1.0 (1.0, 2.0)	
Range	0.0, 4.0	1.0, 3.0	0.0, 4.0	
**Time since Last Procedure**				0.87[2]
N (Missing)	131 (1)	73 (0)	204 (1)	
Mean (SD)	130.8 (87.77)	134.7 (89.77)	132.2 (88.29)	
Median (IQR)	117.0 (67.0, 168.0)	122.0 (63.0, 185.0)	117.5 (66.0, 178.0)	
Range	1.0, 579.0	11.0, 454.0	1.0, 579.0	
**Number of RAIs before Survey**				0.10[2]
N (Missing)	94 (38)	45 (28)	139 (66)	
Mean (SD)	1.3 (0.78)	1.4 (0.84)	1.3 (0.80)	
Median (IQR)	1.0 (1.0, 1.0)	1.0 (1.0, 2.0)	1.0 (1.0, 1.0)	
Range	0.0, 5.0	1.0, 5.0	0.0, 5.0	
**Time since Last RAI**				0.56[2]
N (Missing)	91 (41)	45 (28)	136 (69)	
Mean (SD)	181.5 (203.89)	169.6 (206.41)	177.6 (204.04)	
Median (IQR)	146.0 (104.0, 204.0)	154.0 (72.0, 203.0)	149.0 (100.0, 203.5)	
Range	17.0, 1432.0	14.0, 1423.0	14.0, 1432.0	
**T-Stage**, n (%)				0.37[3]
T1	62 (48.4%)	35 (47.9%)	97 (48.3%)	
T2	39 (30.5%)	17 (23.3%)	56 (27.9%)	
T3/T4	27 (21.1%)	21 (28.8%)	48 (23.9%)	
Missing	4	0	4	
**N-Stage**, n (%)				0.55[3]
N0/Nx	79 (60.3%)	48 (65.8%)	127 (62.3%)	
N1a	22 (16.8%)	13 (17.8%)	35 (17.2%)	
N1b	30 (22.9%)	12 (16.4%)	42 (20.6%)	
Missing	1	0	1	

**M-Stage**, n (%)				1.00[4]
M0	124 (94.7%)	70 (95.9%)	194 (95.1%)	
M1	7 (5.3%)	3 (4.1%)	10 (4.9%)	
Missing	1	0	1	
**8th Stage**, n (%)				0.70[4]
1	114 (87.0%)	66 (91.7%)	180 (88.7%)	
2	12 (9.2%)	5 (6.9%)	17 (8.4%)	
3	2 (1.5%)	1 (1.4%)	3 (1.5%)	
4b	3 (2.3%)	0 (0.0%)	3 (1.5%)	
Missing	1	1	2	
**Gender**, n (%)				0.49[3]
Female	110 (83.3%)	58 (79.5%)	168 (82.0%)	
Male	22 (16.7%)	15 (20.5%)	37 (18.0%)	
**Age at Survey**				0.08[1]
N (Missing)	132 (0)	73 (0)	205 (0)	
Mean (SD)	54.4 (13.19)	51.1 (12.22)	53.2 (12.92)	
Median (IQR)	55.5 (44.0, 64.5)	50.0 (42.0, 61.0)	54.0 (43.0, 64.0)	
Range	23.0, 85.0	27.0, 80.0	23.0, 85.0	
**ATA Recurrence Risk**, n (%)				0.97[3]
Low	74 (56.9%)	42 (57.5%)	116 (57.1%)	
Intermediate	44 (33.8%)	25 (34.2%)	69 (34.0%)	
High	12 (9.2%)	6 (8.2%)	18 (8.9%)	
Missing	2	0	2	
**Experience Recurrences or Required Additional Treatment**, n (%)				0.91[3]
No	96 (73.3%)	54 (74.0%)	150 (73.5%)	
Yes	35 (26.7%)	19 (26.0%)	54 (26.5%)	
Missing	1	0	1	
**ATA Response to Therapy**, n (%)				0.64[4]
Excellent	87 (66.4%)	46 (63.0%)	133 (65.2%)	
Indeterminate	35 (26.7%)	20 (27.4%)	55 (27.0%)	
Biochemical incomplete	4 (3.1%)	5 (6.8%)	9 (4.4%)	
Structurally incomplete	5 (3.8%)	2 (2.7%)	7 (3.4%)	
Missing	1	0	1	
**TSH**, n (%)				**<.01**[3]
A. <0.1	11 (8.4%)	15 (20.5%)	26 (12.7%)	
B. 0.1-0.49	42 (32.1%)	16 (21.9%)	58 (28.4%)	
C. 0.5-2.0	44 (33.6%)	32 (43.8%)	76 (37.3%)	
D. >2.0	34 (26.0%)	10 (13.7%)	44 (21.6%)	
Missing	1	0	1	
**TSH**				0.13[2]
N (Missing)	131 (1)	73 (0)	204 (1)	
Mean (SD)	3.8 (18.86)	1.3 (1.97)	2.9 (15.19)	
Median (IQR)	0.8 (0.3, 2.1)	0.8 (0.2, 1.4)	0.8 (0.3, 1.8)	
Range	0.0, 182.3	0.0, 9.5	0.0, 182.3	
**Thyroid Hormone Therapy**, n (%)				0.55[3]
Levothyroxine monotherapy	120 (91.6%)	65 (89.0%)	185 (90.7%)	

Levothyroxine and liothyronine both as separate prescriptions	11 (8.4%)	8 (11.0%)	19 (9.3%)	
Missing	1	0	1	
**TG**, n (%)				0.29[4]
A. 0-0.19	71 (65.7%)	38 (58.5%)	109 (63.0%)	
B. 0.2-0.99	23 (21.3%)	15 (23.1%)	38 (22.0%)	
C. 1.0-4.99	9 (8.3%)	10 (15.4%)	19 (11.0%)	
D. 5.0-9.99	0 (0.0%)	1 (1.5%)	1 (0.6%)	
E. 10.0+	5 (4.6%)	1 (1.5%)	6 (3.5%)	
Missing	24	8	32	
**TG**				0.28[2]
N (Missing)	108 (24)	65 (8)	173 (32)	
Mean (SD)	52.9 (437.19)	1.2 (4.81)	33.5 (345.75)	
Median (IQR)	0.1 (0.1, 0.4)	0.1 (0.1, 0.6)	0.1 (0.1, 0.5)	
Range	0.1, 4440.0	0.1, 38.5	0.1, 4440.0	
**Presence of TG ab**, n (%)				0.49[4]
No	124 (94.7%)	71 (97.3%)	195 (95.6%)	
Yes	7 (5.3%)	2 (2.7%)	9 (4.4%)	
Missing	1	0	1	
**Sialadenitis**, n (%)				0.40[3]
No	107 (81.7%)	63 (86.3%)	170 (83.3%)	
Yes	24 (18.3%)	10 (13.7%)	34 (16.7%)	
Missing	1	0	1	
**Tear Duct Blockage or Tearing**, n (%)				0.10[4]
No	122 (93.1%)	72 (98.6%)	194 (95.1%)	
Yes	9 (6.9%)	1 (1.4%)	10 (4.9%)	
Missing	1	0	1	
**Dry Mouth**, n (%)				0.06[3]
No	103 (78.6%)	65 (89.0%)	168 (82.4%)	
Yes	28 (21.4%)	8 (11.0%)	36 (17.6%)	
Missing	1	0	1	
**Neck Pain**, n (%)				0.35[4]
No	129 (98.5%)	70 (95.9%)	199 (97.5%)	
Yes	2 (1.5%)	3 (4.1%)	5 (2.5%)	
Missing	1	0	1	
**Hoarseness or Voice Changes**, n (%)				0.36[3]
No	123 (93.9%)	66 (90.4%)	189 (92.6%)	
Yes	8 (6.1%)	7 (9.6%)	15 (7.4%)	
Missing	1	0	1	
**Vocal Cord Paralysis**, n (%)				0.13[4]
No	130 (99.2%)	70 (95.9%)	200 (98.0%)	
Yes	1 (0.8%)	3 (4.1%)	4 (2.0%)	
Missing	1	0	1	
**Hypoparathyroidism**, n (%)				0.92[3]
No	119 (90.8%)	66 (90.4%)	185 (90.7%)	
Yes	12 (9.2%)	7 (9.6%)	19 (9.3%)	

Missing	1	0	1	

[1]Equal variance two sample t-test; [2]Wilcoxon rank sum p-value; [3]Chi-Square p-value; [4]Fisher Exact p-value;

Supplementary Table S2: PSQI: Comparison of clinical characteristics at first survey between patients who completed the baseline survey only versus the baseline and follow-up survey

	Number of Surveys			
	1 (N=92)	2 (N=132)	Total (N=224)	P-value
**Age at Diagnosis**				0.12[1]
N (Missing)	84 (8)	126 (6)	210 (14)	
Mean (SD)	44.2 (14.99)	41.0 (13.98)	42.3 (14.44)	
Median (IQR)	42.7 (35.5, 57.8)	40.4 (30.3, 50.5)	41.3 (31.4, 52.7)	
Range	17.6, 85.0	12.2, 78.3	12.2, 85.0	
**Time since First Diagnosis**				**0.02**[2]
N (Missing)	92 (0)	132 (0)	224 (0)	
Mean (SD)	90.4 (91.03)	67.1 (84.70)	76.7 (87.91)	
Median (IQR)	65.5 (24.0, 131.5)	49.5 (6.5, 90.5)	52.5 (17.5, 106.0)	
Range	0.0, 529.0	-113.0, 431.0	-113.0, 529.0	
**Number of Procedures before Survey**				0.59[2]
N (Missing)	92 (0)	132 (0)	224 (0)	
Mean (SD)	1.3 (0.81)	1.2 (0.71)	1.3 (0.76)	
Median (IQR)	1.0 (1.0, 2.0)	1.0 (1.0, 2.0)	1.0 (1.0, 2.0)	
Range	0.0, 4.0	0.0, 3.0	0.0, 4.0	
**Time since Last Procedure**				0.05[2]
N (Missing)	85 (7)	115 (17)	200 (24)	
Mean (SD)	89.4 (82.86)	73.7 (80.13)	80.4 (81.47)	
Median (IQR)	67.0 (33.0, 119.0)	54.0 (23.0, 92.0)	59.5 (24.0, 104.0)	
Range	1.0, 463.0	0.0, 430.0	0.0, 463.0	
**Number of RAIs before Survey**				0.81[2]
N (Missing)	58 (34)	97 (35)	155 (69)	
Mean (SD)	1.1 (0.82)	1.2 (0.94)	1.1 (0.90)	
Median (IQR)	1.0 (1.0, 1.0)	1.0 (1.0, 1.0)	1.0 (1.0, 1.0)	
Range	0.0, 5.0	0.0, 5.0	0.0, 5.0	
**Time since Last RAI**				**0.01**[2]
N (Missing)	52 (40)	78 (54)	130 (94)	
Mean (SD)	130.4 (199.23)	94.4 (174.11)	108.8 (184.67)	
Median (IQR)	94.5 (44.0, 145.5)	50.5 (23.0, 94.0)	62.0 (30.0, 134.0)	
Range	1.0, 1424.0	1.0, 1423.0	1.0, 1424.0	
**T-Stage**, n (%)				0.54[3]
T1	45 (50.6%)	56 (43.1%)	101 (46.1%)	
T2	24 (27.0%)	39 (30.0%)	63 (28.8%)	
T3/T4	20 (22.5%)	35 (26.9%)	55 (25.1%)	
Missing	3	2	5	
**N-Stage**, n (%)				0.97[3]
N0/Nx	55 (60.4%)	81 (61.4%)	136 (61.0%)	
N1a	17 (18.7%)	23 (17.4%)	40 (17.9%)	
N1b	19 (20.9%)	28 (21.2%)	47 (21.1%)	

Missing	1	0	1	
**M-Stage**, n (%)				0.74[4]
M0	86 (94.5%)	127 (96.2%)	213 (95.5%)	
M1	5 (5.5%)	5 (3.8%)	10 (4.5%)	
Missing	1	0	1	
**8th Stage**, n (%)				0.09[4]
1	80 (87.9%)	117 (89.3%)	197 (88.7%)	
2	6 (6.6%)	11 (8.4%)	17 (7.7%)	
3	1 (1.1%)	3 (2.3%)	4 (1.8%)	
4b	4 (4.4%)	0 (0.0%)	4 (1.8%)	
Missing	1	1	2	
**Gender**, n (%)				0.99[3]
Female	76 (82.6%)	109 (82.6%)	185 (82.6%)	
Male	16 (17.4%)	23 (17.4%)	39 (17.4%)	
**Age at Survey**				**<.01**[1]
N (Missing)	92 (0)	132 (0)	224 (0)	
Mean (SD)	50.9 (13.53)	45.7 (13.14)	47.8 (13.52)	
Median (IQR)	50.0 (42.5, 62.5)	44.5 (36.0, 55.0)	47.0 (38.0, 57.0)	
Range	23.0, 85.0	19.0, 80.0	19.0, 85.0	
**ATA Recurrence Risk**, n (%)				0.41[3]
Low	49 (53.8%)	75 (56.8%)	124 (55.6%)	
Intermediate	36 (39.6%)	43 (32.6%)	79 (35.4%)	
High	6 (6.6%)	14 (10.6%)	20 (9.0%)	
Missing	1	0	1	
**Experience Recurrences or Required Additional Treatment**, n (%)				0.45[3]
No	69 (75.8%)	94 (71.2%)	163 (73.1%)	
Yes	22 (24.2%)	38 (28.8%)	60 (26.9%)	
Missing	1	0	1	
**ATA Response to Therapy**, n (%)				0.66[4]
Excellent	56 (61.5%)	78 (59.1%)	134 (60.1%)	
Indeterminate	25 (27.5%)	36 (27.3%)	61 (27.4%)	
Biochemical incomplete	5 (5.5%)	13 (9.8%)	18 (8.1%)	
Structurally incomplete	5 (5.5%)	5 (3.8%)	10 (4.5%)	
Missing	1	0	1	
**TSH**, n (%)				0.89[3]
A. <0.1	17 (18.7%)	28 (21.2%)	45 (20.2%)	
B. 0.1-0.49	26 (28.6%)	41 (31.1%)	67 (30.0%)	
C. 0.5-2.0	27 (29.7%)	34 (25.8%)	61 (27.4%)	
D. >2.0	21 (23.1%)	29 (22.0%)	50 (22.4%)	
Missing	1	0	1	
**TSH**				0.52[2]
N (Missing)	91 (1)	132 (0)	223 (1)	
Mean (SD)	4.6 (21.74)	9.5 (32.73)	7.5 (28.81)	
Median (IQR)	0.7 (0.2, 1.9)	0.4 (0.1, 1.6)	0.5 (0.1, 1.6)	
Range	0.0, 182.3	0.0, 173.8	0.0, 182.3	
**Thyroid Hormone Therapy**, n (%)				0.32[3]

Levothyroxine monotherapy	80 (87.9%)	122 (92.4%)	202 (90.6%)	
Levothyroxine and liothyronine both as separate prescriptions	10 (11.0%)	10 (7.6%)	20 (9.0%)	
Combined levothyroxine/liothyronine therapy (armour, naturethroid, np thyroid etc)	1 (1.1%)	0 (0.0%)	1 (0.4%)	
Missing	1	0	1	
**TG**, n (%)				0.22[4]
A. 0-0.19	45 (61.6%)	66 (55.9%)	111 (58.1%)	
B. 0.2-0.99	13 (17.8%)	30 (25.4%)	43 (22.5%)	
C. 1.0-4.99	7 (9.6%)	17 (14.4%)	24 (12.6%)	
D. 5.0-9.99	2 (2.7%)	2 (1.7%)	4 (2.1%)	
E. 10.0+	6 (8.2%)	3 (2.5%)	9 (4.7%)	
Missing	19	14	33	
**TG**				0.69[2]
N (Missing)	73 (19)	118 (14)	191 (33)	
Mean (SD)	67.6 (519.94)	9.8 (92.02)	31.9 (329.32)	
Median (IQR)	0.1 (0.1, 0.6)	0.1 (0.1, 0.6)	0.1 (0.1, 0.6)	
Range	0.1, 4440.0	0.0, 999.0	0.0, 4440.0	
**Presence of TG ab**, n (%)				0.74[4]
No	86 (94.5%)	127 (96.2%)	213 (95.5%)	
Yes	5 (5.5%)	5 (3.8%)	10 (4.5%)	
Missing	1	0	1	
**Sialadenitis**, n (%)				0.70[3]
No	74 (81.3%)	110 (83.3%)	184 (82.5%)	
Yes	17 (18.7%)	22 (16.7%)	39 (17.5%)	
Missing	1	0	1	
**Tear Duct Blockage or Tearing**, n (%)				0.76[4]
No	86 (94.5%)	126 (95.5%)	212 (95.1%)	
Yes	5 (5.5%)	6 (4.5%)	11 (4.9%)	
Missing	1	0	1	
**Dry Mouth**, n (%)				0.34[3]
No	72 (79.1%)	111 (84.1%)	183 (82.1%)	
Yes	19 (20.9%)	21 (15.9%)	40 (17.9%)	
Missing	1	0	1	
**Neck Pain**, n (%)				0.45[4]
No	87 (95.6%)	129 (97.7%)	216 (96.9%)	
Yes	4 (4.4%)	3 (2.3%)	7 (3.1%)	
Missing	1	0	1	
**Hoarseness or Voice Changes**, n (%)				0.69[3]
No	86 (94.5%)	123 (93.2%)	209 (93.7%)	
Yes	5 (5.5%)	9 (6.8%)	14 (6.3%)	
Missing	1	0	1	
**Vocal Cord Paralysis**, n (%)				0.15[4]
No	91 (100.0%)	128 (97.0%)	219 (98.2%)	
Yes	0 (0.0%)	4 (3.0%)	4 (1.8%)	
Missing	1	0	1	

**Hypoparathyroidism**, n (%)				0.17[3]
No	85 (93.4%)	116 (87.9%)	201 (90.1%)	
Yes	6 (6.6%)	16 (12.1%)	22 (9.9%)	
Missing	1	0	1	

[1]Equal variance two sample t-test; [2]Wilcoxon rank sum p-value; [3]Chi-Square p-value; [4]Fisher Exact p-value;

Supplementary Table S3: ATA 2015 Risk of Recurrence Criteria

Category	Criteria
ATA low risk	Papillary thyroid cancer (with all of the following):<br>• No local or distant metastases;<br>• All macroscopic tumor has been resected<br>• No tumor invasion of loco-regional tissues or structures<br>• The tumor does not have aggressive histology (e.g., tall cell, hobnail variant, columnar cell carcinoma)<br>• If 131I is given, there are no RAI-avid metastatic foci outside the thyroid bed on the first posttreatment whole-body RAI scan<br>• No vascular invasion<br>• Clinical N0 or ⩽5 pathologic N1 micrometastases (<0.2 cm in largest dimension)<br>Intrathyroidal, encapsulated follicular variant of papillary thyroid cancer<br>Intrathyroidal, well differentiated follicular thyroid cancer with capsular invasion and no or minimal (<4 foci) vascular invasion<br>Intrathyroidal, papillary microcarcinoma, unifocal or multifocal, including BRAFV600E mutated (if known)
ATA intermediate risk	Microscopic invasion of tumor into the perithyroidal soft tissues<br>RAI-avid metastatic foci in the neck on the first posttreatment whole-body RAI scan<br>Aggressive histology (e.g., tall cell, hobnail variant, columnar cell carcinoma)<br>Papillary thyroid cancer with vascular invasion<br>Clinical N1 or >5 pathologic N1 with all involved lymph nodes <3 cm in largest dimension<br>Multifocal papillary microcarcinoma with ETE and BRAFV600E mutated (if known)
ATA high risk	Macroscopic invasion of tumor into the perithyroidal soft tissues (gross ETE)<br>Incomplete tumor resection<br>Distant metastases<br>Postoperative serum thyroglobulin suggestive of distant metastases<br>Pathologic N1 with any metastatic lymph node ⩾3 cm in largest dimension<br>Follicular thyroid cancer with extensive vascular invasion (> 4 foci of vascular invasion)

Supplementary Table S4: ATA 2015 Response to Therapy Criteria

Category	Definition
Excellent Response	Negative imaging<br>and either<br>Suppressed Tg <0.2 ng/mL<br>or<br>TSH-stimulated Tg <1 ng/mL

Biochemical incomplete response	Negative imaging<br>and<br>Suppressed Tg ⩾1 ng/mL<br>or<br>Stimulated Tg ⩾10 ng/mL<br>or<br>Rising anti-Tg antibody levels
Structural incomplete response	Structural or functional evidence of disease<br>With any Tg level<br>With or without anti-Tg antibodies
Indeterminate response	Nonspecific findings on imaging studies<br>Faint uptake in thyroid bed on RAI scanning<br>Nonstimulated Tg detectable, but <1 ng/mL<br>Stimulated Tg detectable, but <10 ng/mL<br>or<br>Anti-Tg antibodies stable or declining in the absence of structural or functional disease
